# French Hospital

**Published:** 1902-03-22

**Authors:** 


### FRENCH HOSPITAL.

Only one governor—a lady—in addition to the committee attended the annual meeting of the French Hospital in Shaftesbury Avenue on the 13th inst. The proceedings, which were as usual conducted in French, were brief, quiet, and businesslike. Dr. A. Vintras, the senior physician, presided, and moved the adoption of the report, which was read by the Treasurer, and adopted without comment. The number of in-patients treated during the year was 820, as compared with 875 in 1900, the difference being caused by the fact that during August and September, 1901, the hospital was closed for repairs. The out-patients numbered 4,667, as against 3,611 in 1900. The ordinary income was £4,045, plus a legacy of £1,590. The ordinary expenditure was £4,064, the extraordinary expenditure being set down as £521, though the outstanding liability for the repairs in course of completion would, if added, have made this item about £1,200. Reference was made to the death of Sir William McCormac, who was for 23 years surgeon-in-chief to the hospital and a member of the committee of management. A brass tablet is to be placed in one of the surgical wards to the memory of the distinguished surgeon, whose promotion to the rank of Commander in the Legion of Honour, of which he had been a Knight for 20 years and an officer for 15 years, gave him so much pleasure when that coveted distinction was conferred on him in 1900. Mr. Edmund Owen, of St. Mary's Hospital, has been appointed surgeon and member of the committee in his place. Pleasure was expressed that His Majesty had acceeded, in most gracious terms, to the request to confer his patronage in succession to the late Queen, and in continuance of his own as Prince of Wales.

